# A Novel *Sphingomonas* sp. Isolated from Argan Soil for the Polyhydroxybutyrate Production from Argan Seeds Waste

**DOI:** 10.3390/polym15030512

**Published:** 2023-01-18

**Authors:** Amina Aragosa, Benedetta Saccomanno, Valeria Specchia, Mariaenrica Frigione

**Affiliations:** 1 Department of Biological and Environmental Sciences and Technologies, University of Salento, 73100 Lecce, Italy; 2 School of Science and Engineering, Al Akhawayn University, Ifrane 53000, Morocco; 3 Department of Innovation Engineering, University of Salento, 73100 Lecce, Italy

**Keywords:** polyhydroxybutyrate, bio-based polymers, argan seeds waste, *Shingomonas*, culture optimization

## Abstract

Polyhydroxybutyrate (PHB) is a biodegradable bio-based polymer synthesized by microorganisms under unfavorable conditions from agro-industrial residues as a source of carbon. These aspects make the bio-based polymer attractive for the mass production of biodegradable plastics, and a definitive replacement for petroleum-based plastics. The aim of this work was to characterize the putative PHB-producing bacterium 1B isolated from the argan soil, to identify the polymer produced, and quantify the PHB production using argan seeds waste. DNA extraction, PCR, and Sanger sequencing were conducted for the molecular identification of strain 1B; the residual biomass and the PHB quantification were measured and compared in the presence of simple sugars and pretreated argan seeds waste. The 1B growth and PHB synthesis were optimized by selecting physical and nutritional parameters: temperature, incubation time, pH, NaCl concentration, and nitrogen sources concentrations. A preliminary characterization of the bio-based polymer extracted was conducted by UV-Visible spectrophotometry and FTIR analysis. The strain 1B was identified as belonging to the genus *Sphingomonas.* The PHB final yield was higher in a growth culture enriched with argan waste (3.06%) than with simple sugars. The selected conditions for the bacterial optimal growth incremented the PHB final yield to 6.13%, while the increase in the argan residue concentration from 1 to 3% in a larger culture volume led to the PHB final yield of 8.16%. UV-Visible spectrophotometry of the extracted sample reported a remarkable peak at 248 nm, as well as FTIR spectra analysis, showed peaks at 1728 and 1282 wavenumber/cm. Both preliminary characterizations demonstrated that the extracted sample is the bio-based polymer polyhydroxybutyrate. The results reported in this work reveal how the costless available argan seeds can be used for polyhydroxybutyrate production using a novel *Sphingomonas* species.

## 1. Introduction

The take-off of the industrial and technological societies induced a rapid expansion of global contamination, particularly caused by persistent pollutants, such as toxic heavy metals and synthetic plastics. Nowadays, scientific research is addressing its interest to manufacture innovative materials used to reduce the toxicity of water contaminated by lead, arsenic, and zinc [1,2,3]. Likewise, the use of non-biodegradable petroleum-based plastics is becoming a challenging issue to be addressed rapidly due to the considerable pollution that this waste cause to our ecosystems and the living organisms that inhabit them [4]. Polyhydroxybutyrate represents an example of an eco-friendly bio-based polymer because of its biodegradable properties as well as the possibility to be produced by living things, such as microorganisms, from naturally occurring raw material [4].

Polyhydroxybutyrate is a bio-based polymer with different brittleness and stiffness depending on the level of crystallinity, transition temperature, and structure. Although it has similar mechanical and physical properties to polypropylene, polyhydroxybutyrate can be synthesized by microorganisms, and aerobically and anaerobically degraded. Several microorganisms have been identified to accumulate PHB granules as a response to unbalanced growth conditions, particularly in excess of carbon sources and the limitation of nitrogen or phosphorous [5]. Among the genera *Bacillus* and *Azotobacter*, various wild species have been isolated and identified: *Bacillus megaterium* [6], *Bacillus thurigiensis* [7], *Bacillus cereus* [8], *Azotobacter vinelandii* [9], *Azotobacter chroococcum* [10].

The use of raw material, such as agricultural and industrial wastes, represents an advantage as it is rich in carbon, inexpensive, renewable, and biodegradable [4]. The use of different types of carbon-rich agro-industrial residues was investigated to maximize PHB production at the lowest economic impact. Sugar cane bagasse, corn cob, teff straw [11], banana peel [12], pineapple waste [13], sugar beet juice [14], pea shell slurry [15], sugarcane juice [16], olive mill wastewater [17], date seeds [18], waste frying oil [19], corn cob [20], cheese whey [21], are only some examples of cheap biomass rich in carbon used for the PHB production. From this perspective, the focus of this work was to value one of the copious agricultural wastes produced in Morocco.

The argan tree *Argania spinosa* forms a forest area of 70% of the woodland area in the northeastern region of Morocco that holds more than 20 million argan trees. The argan fruit is characterized by two parts: the pulp that is covered by a skin, and an internal kernel used for oil production, as edible and cosmetics goods. The argan wastes obtained from the kernels for oil production provide cheap feed to cattle and goats. However, recent chemical studies reported its high level of lipids and carbohydrates that value this residue for further uses [22].

Therefore, the aim of this work was to use argan waste as an inexpensive source of carbon for PHB biosynthesis and extraction. Moreover, a putative PHB producer bacterial species isolated from Morocco argan soil [23] was characterized at the genetic level demonstrating, for the first time, how this bacterial strain is able to use argan wastes for the production of PHB bio-based polymer, subsequently analyzed through FTIR analysis. This research paves the way for a deeper chemical evaluation of the quality and potential applications of this bio-based polymer in a circular economy strategy.

## 2. Materials and Methods

### 2.1. DNA Extraction, PCR, and Sanger Sequencing

DNA extraction of strain 1B1 [23] was performed using Invitrogen Easy-DNA gDNA Purification Kit (Thermofisher Scientific™, Waltham, MA, USA) following the manufacturer’s instructions. An amount of 1 μL of extracted DNA was quantified by NanoDrop ND-1000 droplet spectrophotometer (Thermofisher Scientific, Waltham, MA, USA). The amplification of nearly full-length 16S rDNA was performed using universal primers 27F (5′-AGAGTTTGATCCTGGCTCAG-3′) and 1492R (5′-TACGGYTACCTTGTTACGACTT-3′) (provided by BMR Genomics, Padova, Italy) [24]. Two replicate PCR reactions were prepared, containing 1 μL of 30 ng DNA template, 1 μL of 10 μM Primer 27F, 1 μL of 10 μM Primer 1492R, 5 μL of 10× PCR Buffer (-MgCl_2_), 1.5 μL of 50 mM MgCl_2_, 1 μL of 10 mM dNTPs, 0.4 μL of Platinum^®^ Taq Polymerase (5 U μL, Invitrogen™, Waltham, MA, USA) brought up to a final volume of 50 μL with ultra-pure water. PCR amplifications were performed on SimpliAmp™ Thermal Cycler (Applied Biosystems™, Waltham, MA, USA) under the following conditions: 4 min at 94 °C, followed by 40 cycles of 30 s at 94 °C, 30 s at 57 °C and 1 min at 72 °C, and a final extension step at 72 °C for 10 min. Aliquots of 15 μL of each reaction were analyzed on 1% (*w/v*) agarose gel in TAE buffer. An amount of 30 ng of each PCR reaction was sent to BMR Genomics (Padova, Italy) for ExoSAP clean-up and Sanger sequencing with 12.8 pmol of primer 27F. The resulting abi trace files were aligned using Clustal Omega [25], mismatches were corrected based on the Spectropherograms and 100 bp upstream and downstream were trimmed on Benchling software resulting in a consensus sequence of about 600 bp. 

### 2.2. Species Delimitation Analysis

The 16S sequences of all publicly available *Sphinogomonas* strains were downloaded from NCBI and aligned against our isolate using ClustalW in MEGAX v.10.2.6 (Kumar, et al., 2018, https://www.megasoftware.net/, accessed on 1 May 2022). The resulting alignment was trimmed to obtain the same sequence length across all accessions. These alignments were used to construct a maximum likelihood phylogenetic tree with rapid bootstrap (500 replicates) using the general time reversible + gamma (GTR + G) model in RAxml (Randomized Axelerated Maximum Likelihood) version 8.2.12 (Stamatakis A., 2014, https://cme.h-its.org/exelixis/web/software/raxml/, accessed on 1 May 2022). The resulting tree was used to infer species delimitation with the Bayesian implementation of the Poisson tree processes (bPTP) model [26] with 100,000 MCMC generation and 1% burn-in and then annotated with iTOL web software (Letunic and Bork, 2021, https://itol.embl.de/, accessed on 1 May 2022).

### 2.3. Residual Biomass and PHB Quantification

The production of putative polyhydroxybutyrate was carried out under a limiting nitrogen medium, using sterile mineral salt medium (MSM, Merck, Darmstadt, Germany) containing K_2_HPO_4_ (5 g/L), NaSO_4_ (0.5 g/L) MgSO_4_ · 7H_2_O (0.4 g/L), Glucose (20 g/L) and 0.1% of mineral solution containing FeSO_4_ · 7H_2_O (2.8 g/L), MnCl_2_ · 4H_2_O (2 g/L), CoSO_4_ · 7H_2_0 (1.5 g/L), CuCl_2_ · 2H_2_0 (0.2 g/L), and ZnSO_4_ · 7H_2_0 (0.3 g/L) [27] in a 50 mL culture. The culture was prepared by subculturing the 1B strain in 5 mL Luria Bertani broth [28]. After 24 h of incubation at 37 °C, 1 mL of the culture was inoculated in 50 mL mineral salt medium in a 250 mL conical flask at 37 °C for 48 h [11].

#### 2.3.1. Residual Biomass Quantification 

The dry cell weight (DCW) was determined by gravimetric analysis. After incubation, the 50 mL culture was centrifuged at 5000 rpm for 20 min employing the Hettich EBA 30 centrifuge (Labexchange, GmbH, Burladingen, Germany), the pellet was washed twice with distilled water and dried at 100 °C for 24 h to a constant weight [29]. The residual biomass was calculated as a difference between the DCW and the putative PHB extraction to estimate the efficiency in the bio-based polymer production by the isolated strain 1B [30], as reported in following Equation (1):

Residual biomass (g/L) = DCW (g/L) − extracted putative PHB (g/L)(1)

#### 2.3.2. PHB Extraction and Quantification

The putative PHB was extracted from the pellet prepared for the DCW quantification by using the Law and Slepecky method [31]. The cell pellet was extracted from 50 mL culture and was digested with 30% sodium hypochlorite (Sigma-Aldrich, affiliated with Merck, KGaA, Darmstadt, Germany) at 37 °C for 2 h. The mixture was then centrifuged at 5000 rpm for 20 min; the supernatant was discarded, and the pellet washed with distilled water, acetone, and ethanol, sequentially (Sigma-Aldrich, affiliated with Merck, KGaA, Darmstadt, Germany). After centrifugation, the pellet was dissolved in 5 mL hot chloroform (Sigma-Aldrich, affiliated with Merck, KGaA, Darmstadt, Germany) and left overnight for complete evaporation of the solvent at room temperature. The putative PHB accumulation by strain 1B was weighed and recorded. The putative PHB extracted was calculated to estimate the bio-based polymer percent in the dry cell weight [30], according to:

extracted putative PHB (%) = extracted PHB (g/L)/DCW (g/L) × 100(2)

To qualitatively determine the presence of PHB, an immediate stain of the extract was performed using Sudan black staining prepared by dissolving 0.3 µg of powder in 100 mL of 70% ethanol. For the microscopic preparation, the smear of the biopolymer extract was covered with the Sudan black solution on a clean, grease-free glass slide. After staining for 15 min the slide was washed with xylene and observed under a compound light microscope with immersion oil at 100× [32].

**Figure 1:**
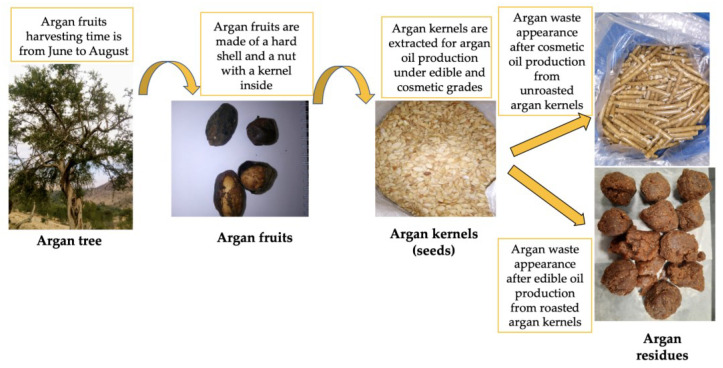
Argan fruits are harvested in a period from June to August. The hard pulp is removed, and the kernels extracted from the nut are pressed for the oil extraction. The argan residue was used as waste biomass for biopolymer extraction.

Additional measurements were carried out to assess the residual biomass and to quantify the bio-based polymer produced in the presence of different carbon sources. Fructose, maltose, saccharose, sorbitol, lactose, and mannose were separately incorporated in the MSM to replace glucose (Fisher Scientific, Goteborg, Sweden). The analyses were repeated by using a pretreated argan pulp, i.e., a residue obtained from the extraction of the oil from the argan, as described in the following paragraph [11]. Argan fruits are made of two parts: the hard mesocarp, and the nut which contains the kernels used for argan oil production. Two argan wastes can be produced: one from cosmetic oil production and the other from edible oil production [33]. In Figure 1, the steps for the argan seeds pulp production starting from the argan fruit harvest till the waste production due to the oil extraction can be appreciated. In this work, argan waste obtained from edible oil production was used for bio-based polymer synthesis and extraction.

#### 2.3.3. Argan Seeds Pulp Pretreatment

The argan seed waste employed in this work was donated by the “Cooperative Feminine Amagour Argan”, one of the argan cooperatives factories located in Taroudant, a region located in southwestern Morocco. In these factories, the workers are employed in the handicraft production of cosmetics and food products such as oils, beauty creams, soap, and shampoo. The waste obtained after these processes was then pretreated in order to obtain the optimal nutrient extraction for bacterial growth and the PHB extraction [34]. To this aim, the argan pulp was dried in an oven (Heratherm™ General Protocol Ovens-230VAC 50/60 Hz, Carthage, MO, USA) at 65 °C for 24 h; it was then ground using a blender (Waring Laboratory Blender, Sigma-Aldrich, St. Louis, MO, USA) at 45,000 rpm for 5 min and, finally, sieved to select only the 0.4 mm ground particles. An amount of 20 g of these particles was first treated using NaOH and Ca(OH)_2_ solutions (Merck, Darmstadt, Germany) (0.5, 1, 2% *w/v*) and then autoclaved at 121 °C for 15 min. The samples were then filtered with Whatman filter paper (N°1, pore size 0.11 µm, Sigma-Aldrich, St. Louis, MO, USA), neutralized with sterile water, and dried overnight at 80 °C employing Heratherm General Protocol Oven. The neutral samples were treated with H_2_SO_4_ and H_3_PO_4_ solutions (Merck, Darmstadt, Germany) (1, 2, 3%, *v/v*), autoclaved (Systec V-40, Systec GmbH, Linden, Germany), and filtered again. The final pH of the obtained sample was, finally, adjusted to pH 7 by adding NaOH [13]. The obtained pretreated argan seeds pulp sample was used to replace glucose in the MSM for the residual biomass and the PHB measurements.

### 2.4. Effect of Growth Medium Optimization on the Bacterial Growth, Dry Cell Weight, and PHB Production

Different growth parameters were selected and implemented to determine the optimal growth conditions of the novel strain 1B. To this aim, 1 mL of each subculture in Luria Bertani was inoculated in a 250 mL conical flask containing 50 mL MSM with 1% of pretreated argan seeds pulp. The effect of temperature (i.e., 20, 24, 28, 32, 36, 40 °C), incubation time (from zero to 72 h, using 12 h intervals), pH (from 2.5 to 10.5, using an interval of 1), NaCl concentrations (from zero to 10%, with 1% interval), different nitrogen sources (i.e., NH_4_Cl, NH_4_NO_3_, (NH_4_)_2_SO_4_, peptone, tryptone, beef extract, and yeast extract), were assessed one at a time for the DCW and PHB production [11]. Spectrophotometric analysis using the spectrophotometer Jenway6320D (Fisher Scientific, Leicestershire, UK) at 600 nm was used to select the highest optical density of the culture [35,36], while the DCW and the putative PHB production were calculated according to Equations (1) and (2). Finally, a growth curve of the bio-based polymer produced from the species 1B with all optimal parameters was constructed and compared to the 1B growth curve without optimal conditions to appreciate the microorganism’s growth enhancement under selected conditions for the bio-based polymer synthesis.

### 2.5. PHB Extraction Using Increasing Argan Seeds Pulp Concentrations and Culture Volume 

Once every single parameter was optimized, a culture growth with all selected parameters was employed to quantify the putative PHB production of species 1B by increasing at first, the residue concentrations, and next the culture fermentation volume [37]. The dry cell weight and the production of the bio-based polymer were quantified by using increasing concentrations of pretreated argan waste (1, 2, 3, 4%, *w/w*) by following the procedure reported in Section 2.3 with all optimal parameters implemented. After selecting the optimal argan residue concentration, the putative PHB final yield was determined in a larger culture medium. An amount of 5 mL of the 1B subculture was transferred in a 1000 mL conical flask with 500 mL MSM enriched with 3% of pretreated argan seeds waste. PHB final yield with different concentrations of argan seeds waste and larger culture volume was measured and reported in the results section.

### 2.6. Preliminary Characterization of the Extracted PHB 

#### 2.6.1. UV-Visible Spectrophotometry

The extracted putative PHB sample of the novel strain 1B was digested with H_2_SO_4_ at 100 °C for 10 min [38]. For a preliminary identification of the bio-based polymer, this product was analyzed at 235 nm in a range of 200 to 800 nm using a UV-Visible spectrophotometer (UV-Visible spectrophotometer, V-530, Jasco, Tokyo, Japan) using H_2_SO_4_ as blank [39].

#### 2.6.2. Chemical Characteristics by FTIR Analysis

The extracted compound, subjected to a lyophilization process in order to completely evaporate the chloroform, was analyzed by an FTIR spectroscopy (JASCO FTIR-6300, Tokyo, Japan) over the range of 4000 to 400 wavenumber/cm, placing the samples on K-Br discs [38]. In this way, it was possible to identify the different functional groups present in the extracted compound in order to confirm that the extracted bio-based polymer was PHB [39].

## 3. Results and Discussion

### 3.1. 16S rDNA Sequencing and Bacterial Identification of 1B Strain

The obtained 16S rDNA sequence was used to infer taxonomic identification through the BlastN software on 1 May 2022 (http://www.ncbi.nlm.nih.gov/BLAST/), revealing that the isolate under study belongs to the genus *Sphingomonas*. In particular, the best hit of the Blast output resulted in a 98.6% identity and 100% query coverage with *Sphingomonas dokdonensis* (MK571193.1), as reported in Figure S1.

**Figure 2:**
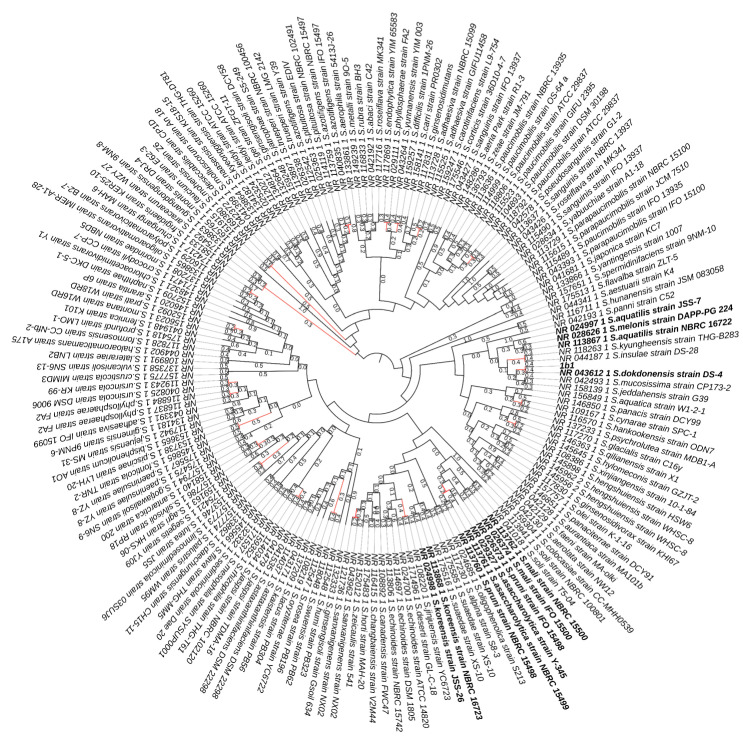
Species delimitation tree generated by the Bayesian Poisson tree processes (bPTP) model, using a fragment of the 16S rDNA. Black lines indicate branching processes among species, red lines indicate branching processes within species. Support values shown on the tree branches represent the posterior probabilities of those taxa forming one species. In bold are represented the tested *Sphingomonas* spp.

To further investigate this result, we applied a coalescent species delimitation method named the Bayesian implementation of the Poisson tree processes (bPTP). This method analyses the number of substitutions between input sequences and assumes that a higher degree of molecular variability is expected between species than within a species [26]. In the present bPTP analysis, the dataset included all the available 16S sequences of the genus *Sphingomonas* corresponding to our sequenced fragment. Even this analysis shows a high degree of similarity between our isolate and *Sphingomonas dokdonensis*, which are considered as one species although with low Bayesian support values (0.377) (Figure 2). The percentage of identity at 98% and the low Bayesian support value indicate that 1B could be a novel species of the *Sphyngomonas* genus.

To support this result, we performed a biochemical comparison of strain 1B with *S. dokdonensis* and other representative *Sphingomonas* species [40]. Table 1 reports the colony and biochemical characteristics of *Sphingomonas* species compared to our strain 1B. Strain 1B already analyzed morphologically and biochemically in the former work published by Aragosa et al. (2022) [23], presents evident biochemical differences in the metabolism of sugars compared to *S. dokdonensis* species. These biochemical dissimilarities, together with the molecular results, support that 1B is a new species of the *Sphyngomonas* genus.

### 3.2. Bio-Based Polymer Extraction and Quantification from 1B Strain by Using Different Carbon Sources

**Figure 3:**
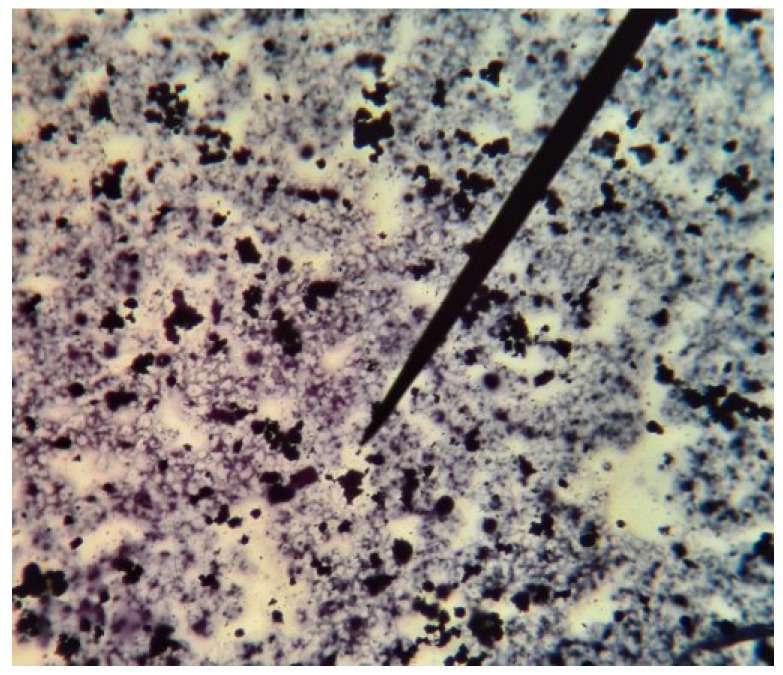
Bio-based polymer extracted from species 1B and stained with Sudan black.

Strain 1B was previously identified as a putative PHB-producing bacterium [23]. With the aim to characterize the bio-based polymer produced in the 1B bacterial cells, we started extraction and quantification experiments as reported in the literature [41]. The experiment was conducted, at first, in 50 mL of MSM culture added with 1% glucose, which is the primary carbon source used in growth media. A confirmation of the bio-based polymer presence was the microscopic observation at 100× of the putative PHB extracted from the MSM culture enriched with glucose and stained with Sudan black (Figure 3). In the figure, the presence of black areas corresponding to the accumulation of the bio-based polymer can be appreciated.

Then, we quantified the extract, as described in the Materials and Methods section, obtaining an average value of 0.57 g/L of bio-based polymer extracted by fermenting 1 g of glucose. To test for improvement of the bio-based polymer amount produced, we replaced glucose in the culture medium with other carbon sources, such as fructose, maltose, saccharose, sorbitol, lactose, and mannose. The measurements for the bio-based polymer final yield were repeated three times for each sugar, and the mean and standard deviation (SD) were calculated [41,42]. Although it was measured with a low putative PHB final yield, it can be appreciated in Table 2 how strain 1B more effectively uses glucose, fructose, and saccharose for the bacterial growth, with a respective bio-based polymer final yield of 0.57 ± 0.06 g/L, 0.54 ± 0.11 g/L, and 0.58 ± 0.22 g/L per 1 g of fermented sugar. As reported in the literature by Sakthiselvan and Madhumathi (2019), when *Sphigomonas* spp. grew on culture media enriched with different sugars, the strains demonstrated better growth, sugar assimilation, and PHB production with disaccharidases and aldohexoses, while failed the assimilation of pentoses, ketoses and starch [43]. The PHB extracted was found to be high with sucrose and mannose, corresponding to 55–60% of the DWC. Compared to these results reported in the literature, our strain 1B also shows a high biopolymer production in the presence of saccharose as well as in the aldohexose, glucose. On the contrary, since 1B shows a high PHB extraction with the presence of ketohexose, and fructose, but not in presence of mannose, the obtained results confirm what was already mentioned in the molecular analysis, so the isolated species from argan soil is a new *Sphingomonas* sp.

### 3.3. Bio-Based Polymer Extraction and Quantification from 1B Strain by Using Argan Seeds Waste in the Culture Medium

As it was already reported in other studies, the use of agricultural and industrial food wastes represents a rich-nutrient alternative for a cost-efficiency bacterial production of PHB [13,18]. We tested, for the first time, the use of argan residues, obtained from the oil extraction, as a carbon source for 1B strain culture. Specifically, argan seeds pulp is a rich-sugar waste that has non-production costs since it is obtained as a waste product from argan oil extraction. We also focused on this source because strain 1B was isolated from Morocco’s argan soil and in the natural context this bacterium can have adapted to use residues of the argan fruit from the soil. Although the chemical composition of argan oil is well known, few studies were reported in the literature to describe the chemical combination of the argan seeds residue, which is used to feed goats and farm animals. According to Harhar et al. (2019), the residue has a high level of palmitic, oleic, linoleic, and linolenic fatty acids, while the groups of most represented sugars are glucose (5.6%), fructose (9.9%), and saccharose (4.6%), and rhamnose (0.6%) [22].

**Figure 4:**
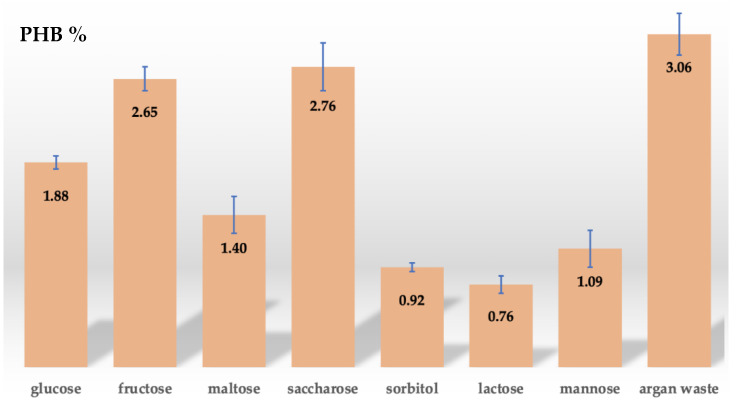
Bio-based polymer final yield extracted from 50 mL 1B culture with 1% of different carbon sources and pretreated argan seeds pulp.

We replaced the sugar source in the culture medium with pretreated argan seeds pulp. The wastes were dried, grounded, filtered, and adjusted to pH 7. 1% of the pretreated sample was added to the 50 mL MSM medium containing 1 mL of 1B strain subculture. After 48 h of incubation at 37 °C the DCW, putative PHB, and residual biomass were measured. The DCW and the putative PHB extracted measured 18.65 ± 0.11 g/L, and 0.57 ± 0.60 g/L, respectively, while the residual biomass was 18.08 g/L. The putative PHB final yield was 3.06%. A comparison of the bio-based polymer yield obtained with the different carbon sources and the argan residue is reported in Figure 4. It can be observed how a higher amount of putative PHB can be obtained by replacing single sugars in the pretreated argan waste. This result indicates that the residue is a rich-sugar waste, containing a combination of simple sugars that are consumed by the species for the synthesis of the polymer.

### 3.4. Optimization of the Bacterial 1B Growth, and Bio-Based Polymer Production

**Figure 5:**
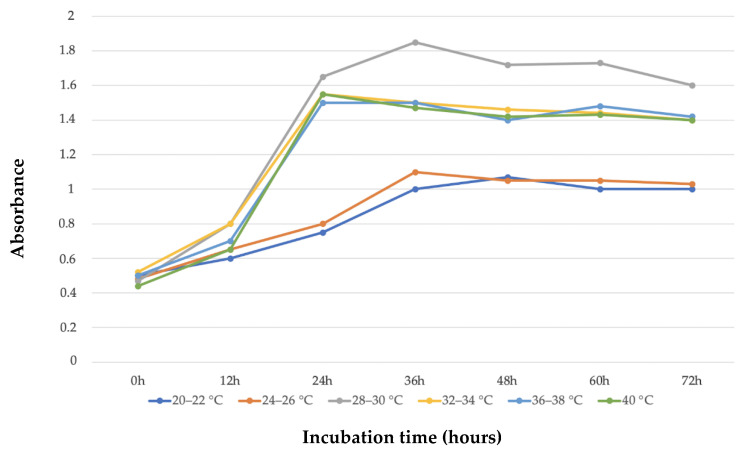
Strain 1B growth at different incubation temperatures and times.

With the aim of incrementing the bio-based polymer production amount from 1B strain, we performed experimental optimization of its growth. To determine the optimal culture conditions of the novel strain 1B, several parameters were selected according to the literature [11,29,37]. The following variables were selected: growth temperature, incubation time, N sources, pH, and NaCl concentrations. Strain 1B was cultivated in 50 mL MSM medium enriched with 1% pretreated argan seeds pulp and incubated at different temperatures, from 20 to 40 °C, and optical density (OD) was determined every 12 h The same setting was carried out, this time at 30 °C, to select the optimal incubation time in a range of 0 to 72 h, with OD measured every 12 h. Strain 1B growth at different temperatures from 0 to 72 h of incubation can be appreciated in Figure 5. Among all selected temperatures, it is possible to identify that the greatest bacterial growth is at 28–30 °C for 36 h of incubation. At these growth conditions, the measurements of DCW and putative PHB extracted were, respectively, 20.34 g/L and 3.25%.

**Figure 6:**
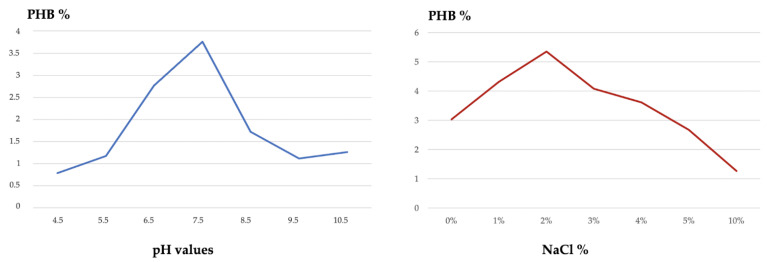
Effect of different pH values and NaCl concentrations on the putative PHB final yield.

Similarly, optimal pH, NaCl concentrations, and N sources were determined, and every time the already selected optimal conditions were maintained in the following selecting parameter to be tested. The favorable pH was determined in a range of 4.5 to 10.5 and DCW and putative PHB was measured at an interval of 1. The novel species showed very low productivity at acidic pH, while the highest putative PHB production corresponds to pH 6.5 and 7.5, measured as 2.77 g/L and 3.76 g/L, respectively. In the following optimization tests, pH 7 was maintained. The optimal sodium chloride concentration was measured from 0% to 10%, with measurements taken every 1% interval. The 1B strain still demonstrated growth and putative PHB production at 0% NaCl (0.61 g/L) and at 10% (0.47 g/L), however, the favorable 2% NaCl allowed a putative PHB extraction of 1.17 g/L, corresponding to a 5.35% of final yield. What is reported so far can be observed in Figure 6 where the graphs of the PHB final yield at different pH values and NaCl concentrations are represented.

**Figure 7:**
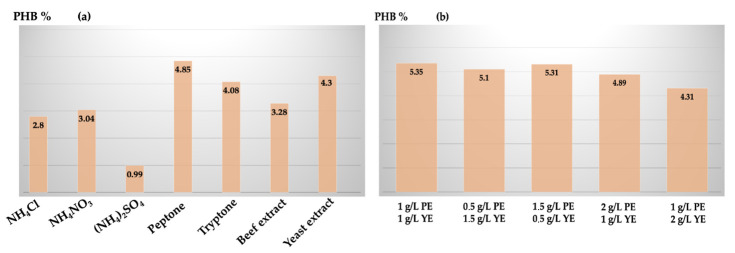
(**a**) Putative PHB final yield by using different nitrogen sources, and (**b**) by using peptone (PE) and yeast extract (YE) as the best nitrogen sources at different ratios.

The final condition to be tested was the different N sources: NH_4_Cl, NH_4_NO_3_, (NH_4_)_2_SO_4_, peptone, tryptone, yeast extract, and beef extract. 0.25% of each N source was added to the MSM enriched with 1% pretreated argan residue, and putative PHB final yield was measured. As can be seen from Figure 7a, the lowest quantity of bio-based polymer was extracted with (NH_4_)_2_SO_4_ enrichment, while the best bio-based polymer productions were appreciated with both, peptone (0.99 g/L) and yeast extract (0.86 g/L). Peptone and yeast extract were both maintained in the culture medium and their different concentration ratios were evaluated (1:1, 1:2, 2:1 *w/w*). The optimal peptone-yeast extract concentration ratio, corresponding to the highest putative PHB production, was 1:1 as can be seen in Figure 7b.

All selected optimal parameters and the relative DCW, bio-based polymer production, and final yield are reported in Table 3, where the gradual increase in bio-based polymer yield can be appreciated for each optimal parameter selected. 

**Figure 8:**
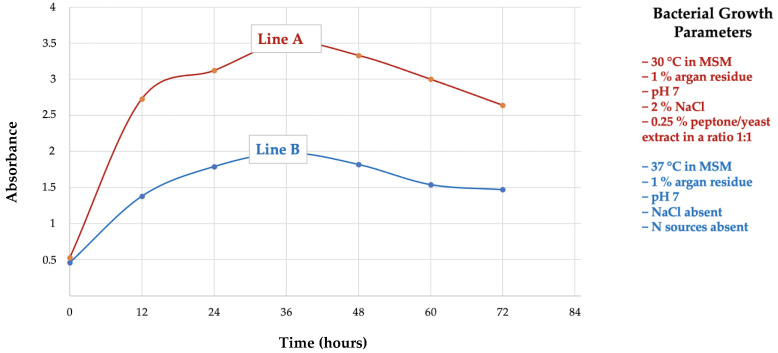
Bacterial growth curve of strain 1B; Line A shows the bacterial growth under selected optimal parameters, Line B shows the bacterial growth under unselected optimal parameters, and in the absence of NaCl and N sources.

Measurements of the optical density (OD) at 600 nm were conducted from the 50 mL culture to estimate if the higher productivity of putative PHB corresponds to higher bacterial growth, too. The bacterial growth, reported in Figure 8, was determined in two different conditions: the first condition (line A), with all selected parameters implemented, and the second condition (line B) with none of the optimal parameters implemented. In the presence of selected optimal parameters, 1B strain grew at 30 °C, in MSM enriched with 1% argan residue, pH 7, 2% of NaCl, and 0.25% of peptone and yeast extract in a 1:1 ratio. With none of the selected optimal parameters implemented, 1B strain grew at 37 °C, in MSM enriched with 1% argan residue, pH 7, and without NaCl and N sources added. As can be observed in Figure 8, the strain shows a greater growth at 30 °C with NaCl and N sources added (line A). This result is another evidence that the greater the bacterial growth, the higher the amount of bio-based polymer synthesized by the microorganism, and, consequently, the higher will be the quantity of bio-based polymer extracted from the culture.

It was already mentioned earlier how the 1B strain has high 16s sequence homology with the *Sphingomonas dokdonensis* species. Moreover, the study of the optimal growth culture conditions also shows some evident analogies between the two species, such as the optimal growing temperature, pH, and NaCl concentration [44]. This interpretation confirms that the novel isolated species belongs to the genus *Sphingomonas* and is closely related to the species *Sphingomonas dokdonensis*.

### 3.5. Improvement of Putative PHB Extraction Using Increasing Argan Residue Concentrations and Culture Volume

The next step in the study of the bacterial productivity for the maximization of putative PHB final yield was to evaluate the bacterial growth using increasing concentrations of the residue and fermentation volumes. Nutritional and physical growth parameters were selected in a 50 mL MSM with 1 g/L of pretreated residue. Supplementary fermentations were conducted in the same volume but by increasing the concentration of the residue to 2 g/L, 3 g/L, 4 g/L, and 5 g/L. As showed in Table 4, the maximum putative PHB extraction in a 50 mL culture was possible with a supplement of 3 g/L of the residue. Concentrations higher than 3 g/L, even though resulted to a great DCW extractions, leads to a very low putative PHB production.

These results are in accordance with what was reported by Saleem et al., (2014), where increasing PHB final yields were observed by increasing the substrate concentration from 1 to 3%, with a maximum of extraction at 3% using maltose as the best source of carbon, while a decrease in PHB extraction was noticed starting from 4% [37]. By performing statistical analysis for the groups of data obtained from the conditions tested and reported in Table 4, it was determined that all groups of data were consideringly different with a significance higher than 96%. This supports what is already found with the means, so the results obtained are significant, and an increase in residue concentration corresponds to an increase in PHB final yield. In discordance was the analysis for the PHB final yield in the groups of data for 4 g and 5 g of argan residue. In these last groups, the *p*-Value was higher than 0.05 (t_(10)_ = 0.211) leading to a significance lower than 80%, which corresponds to a high probability that data are random and results cannot be scientifically accepted for these two groups of data.

**Figure 9:**
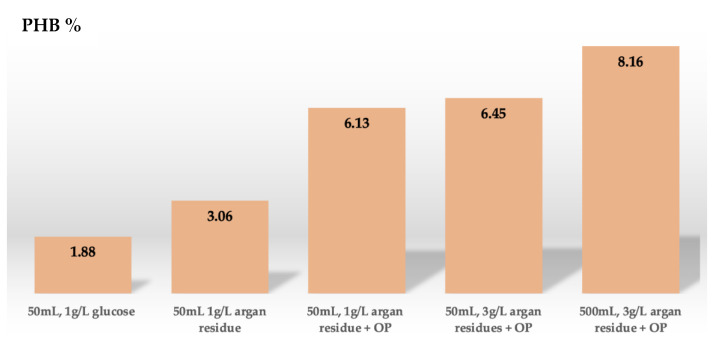
Progressive increase in putative PHB final yield throughout this work.

The fermentation volume is another important variable to increment the bio-based polymer amount extracted from bacterial culture. We performed extraction experiments starting from 500 mL of bacterial culture, respecting all the nutritional and physical conditions selected previously. In these conditions, the DCW and putative PHB produced were 23.43 g/L and 1.92 g/L, respectively, while the putative PHB final yield was calculated as 8.16%. This value is higher compared to the 6.13% obtained in the 50 mL culture, which explains the importance of volumes for more efficient production of the bio-based polymer by the bacterial strain. Carofiglio et al. (2015) valorized the use of toxic oil mill wastewater for the extraction of PHB (31.4 mg/L) using a 500 mL volume culture [17]. Again, the efficiency of *Pseudomonas* sp. RZS1 for PHB final yield (20 µg/mL) was determined in 100 mL volume [38], or when for the first time the pretreated cardboard industry wastewater was used in 250 mL culture to extract 5.236 g/L of PHB [30]. The progressive increase in bio-based polymer final yield throughout this work can be appreciated in Figure 9, where the putative PHB percent was maximized gradually by replacing 1 g/L of glucose (1.88% of bio-based polymer) with 1 g/L of argan waste (3.06% of bio-based polymer), selecting optimal culture conditions (6.13% of bio-based polymer), increasing the argan residue from 1 g/L to 3 g/L (6.45% of bio-based polymer), and boost up the fermentation from 50 mL to 500 mL culture volume (8.16% of bio-based polymer). However, after looking at the means of what was obtained from the performed tests it was necessary to evaluate the significance of this increase in PHB % in all tested conditions reported earlier. The difference in PHB % between 50 mL culture + 1 g/L glucose (M = 1.88; SD = 0.61) and 50 mL culture + 1 g/L argan residue (M = 3.06; SD = 0.23) was extremely significant with a *p*-Value lower than 0.05 (t_(10)_ = 0.017) meaning that less than 2% are random data and more than 98% are consideringly different data. The difference in PHB % between 50 mL culture + 1 g/L of argan residue (M = 3.06; SD = 0.23) and 50 mL culture + 1 g/L argan residue in presence of optimal parameters (M = 6.13; SD = 0.69) showed a *p*-Value of 0.008, once again showing that more than 99% of data are significant. On the contrary, *p*-Value for the PHB % difference between 50 mL culture + 1 g/L argan residue in presence of optimal parameters (M = 6.13; SD = 0.69) and 50 mL culture + 3 g/L argan residue in presence of optimal parameters (M = 6.45; SD = 0.77) showed a *p*-Value of 0.329 demonstrating that data are just random and not significant when the argan residue concentration is increased from 1 to 3 g/L. Finally, the last condition to test the difference in PHB % between 50 mL culture + 3 g/L argan residue in presence of optimal parameters (M = 6.45; SD = 0.77) and 500 mL culture +3 g/L argan residue in presence of optimal parameters (M = 8.16; SD = 0.12) reported a significance higher than 97% with a *p*-Value of 0.020, so there is a high confidence that data are consideringly different. Overall, the statistical analysis shows that the obtained results in terms of PHB final yield for most of the tested conditions exhibits a significance higher than 95%, so the increasing production of PHB already observed with the means can be accepted.

### 3.6. Preliminary Characterization of the Extracted Bio-Based Polymer

#### 3.6.1. UV-Vis Spectrophotometry 

**Figure 10:**
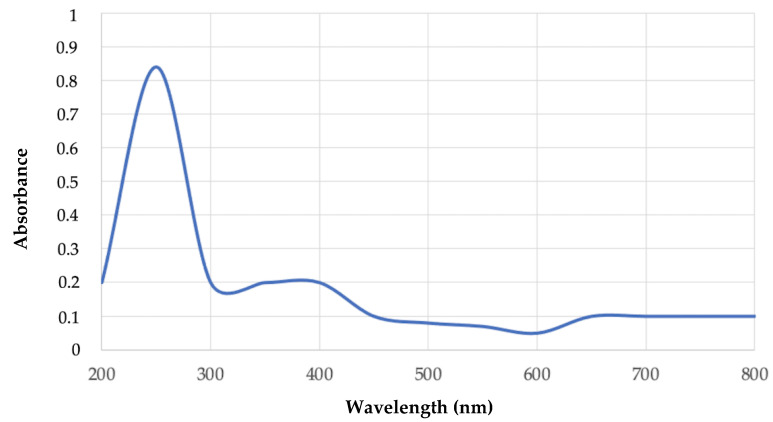
UV-Visible spectrum of putative PHB extracted from strain 1B.

The putative PHB extracted sample was used for preliminary identification of the bio-based polymer in UV-Visible spectrophotometry, in a range of 200 to 800 nm. The spectrum obtained by this analysis is reported in Figure 10. For the extracted compound a peak at 248 nm was observed, which was absent in the control absorbance spectrum. This peak is reported to be characteristic of the PHB polymer as previously reported [38,39]. This result strongly supports the presence of PHB in the extracted material.

#### 3.6.2. FTIR Analysis

**Figure 11:**
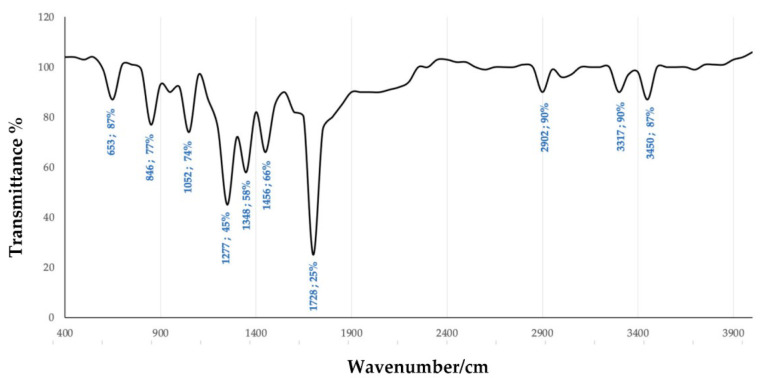
FTIR spectrum of putative PHB extracted from strain 1B.

The FTIR analysis for the identification of the chemical groups characterizing the extracted compound was carried out, recording the IR spectra in the range from 400 to 4000 wavenumber/cm. The results of this analysis were compared with the spectra found for a standard PHB, reported by Lathwal et al. (2018) [45]. In Figure 11, the peaks at 1728 and 1277 wavenumber/cm, corresponding to the C=O ester group and CH group, respectively, are observed: these two peaks are those characteristics of the PHB polymer [5,46,47]. This result confirms that hydroxybutyrate polymer is present in the product extracted from strain 1B. Other relevant peaks of absorption observed in the analyzed sample were found to match those described by Ansari and Fatma (2016) [48]. The sample also exhibits additional peaks (i.e., at 1052, 3317, and 3450 wavenumber/cm) which appear to be incongruous with the standard PHB analysis. The presence of these supplemental peaks, not characteristic of a standard PHB, could be associated with the presence of impurities, such as cell debris or extractive solvents, which persisted after the extraction process. Therefore, the FTIR spectrum of the sample confirms the presence of the PHB polymer but also indicates that a further enhancement of the sample purification during the bio-based polymer extraction would be necessary.

## 4. Conclusions

This study presented the possibility to produce the bio-based polymer PHB from a microorganism, formerly isolated from argan soil, and here identified as belonging to the genus *Sphingomonas.* Moreover, the possibility to use argan seeds waste to produce PHB was evaluated. The microorganisms exhibited an enhanced growth in a culture medium enriched by pretreated argan seeds residue than in single sugars, such as glucose, fructose, lactose, maltose, saccharose, and mannose. More efficiently, the microorganism was able to synthesize PHB granules when physical (temperature, incubation time, and pH) and nutritional (concentrations of NaCl, and nitrogen sources) growth parameters were selected. Once again, the PHB extraction was enhanced by increasing the biomass residue concentrations, and by inducing the microorganism growth in a larger culture volume. Therefore, this work not only identified a novel *Sphingomonas* sp. PHB producer, but also promotes the use of cheaply available raw material, such as argan seeds waste as the sole source of carbon for bio-based polymer production. To follow up on this work, additional molecular aspects of the bacterial strain 1B, such as the identity of genes and promoters responsible for PHB synthesis, and the possibility to produce new recombinant bacterial species, will be considered in the subsequent work to evaluate higher final yields of the bio-based polymer. Further characterization of the bio-based polymer will be necessary to better understand its chemical, physical, and mechanical aspects for its potential uses for the synthesis of the bio-based polymer.

## Figures and Tables

**Table 1:** Correlation of 1B strain with some *Sphingomonas* spp. relative to biochemical characteristics.

	1B Strain Isolated from Argan Crop Soil	Sphingomonas Dokdonensis	Sphingomonas Asaccharolityca	Sphingomonas Mali	Sphingomonas Pruni	Sphingomonas Aquatilis	Sphingomonas Koreensis	Spsphingomonas Mmelonis
Beta galactosidase	+	+	+	+	+	+	+	+
Gelatin hydrolysis	+	+	−	−	−	−	−	−
Arabinose	+	−	+	+	+	+	−	+
Fructose	+	−	−	+	+	ND	ND	+
Galactose	−	−	−	+	+	ND	ND	+
Maltose	+	−	+	+	+	+	+	+
Mannose	+	+	+	+	+	−	−	+
Sucrose	+	−	−	+	+	+	+	+
Gram	−	−	−	−	−	−	−	−
H2S production	−	−	ND	ND	ND	ND	ND	ND
Colony color/morphology	regular white translucent, smooth surface, viscid clustered with 0.5–1 mm in diameter	circular, convex, smooth, glistening, yellow in colour and 0.8–1.0 mm in diameter	Ovoid, yellow pigmented colonies, diameter > 1 mm	Ovoid, yellow pigmented colonies with 1.00–1.5 mm	Yellow colonies with greyish white pigmentations, 0.8–1.0 mm in diameter	Yellow round colonies with darker pigmentations, 0.8–1.0 mm in diameter	Yellow round colonies with darker pigmentations, 1.0–1.2 mm in diameter	Deep yellow round colonies with dark yellow pigmentations, 1.0–1.2 mm in diameter
Chloramphenicol	S	S	S	S	S	ND	S	ND
Gentamicin	IM	S	S	S	S	ND	R	ND
Kanamycin	S	S	S	S	R	ND	R	ND
Penicillin	R	R	S	R	R	ND	R	ND
Ampicillin	R	R	S	S	R	ND	R	ND

Abbreviations: ND, not determined; S, susceptible; R, resistant; IM, intermediate.

**Table 2:** Use of different carbon sources for the extraction of the bio-based polymer.

Carbon Source *	Residual Biomass * g/L	DCW * g/L	Bio-Based Polymer * g/L	Bio-Based Polymer %
glucose	18.08	18.65 ± 0.23	0.57 ± 0.06	1.88
fructose	19.81	20.35 ± 0.44	0.54 ± 0.11	2.65
maltose	15.48	15.70 ± 0.21	0.22 ± 0.17	1.40
saccharose	20.43	21.01 ± 0.05	0.58 ± 0.22	2.76
sorbitol	12.88	13.00 ± 0.10	0.12 ± 0.04	0.92
lactose	13.11	13.21 ± 0.13	0.10 ± 0.08	0.76
mannose	13.63	13.78 ± 0.37	0.15 ± 0.17	1.09

* All results in g/L correspond to the amount extracted from 1 g of sugar in 50 mL culture.

**Table 3:** Gradual increase in DCW and putative PHB production of the species 1B in 50 mL culture with selected optimal growth parameters.

Growth Conditions	Selected Optimal Conditions	DCW g/L	Bio-Based Polymer g/L	Bio-Based Polymer %
Temperature	30 °C	ND	ND	ND
Incubation time	36 h	20.34 ± 0.10	0.67 ± 0.11	3.25
pH	6.5–7.5	20.07 ± 0.13	0.76 ± 0.07	3.76
NaCl	2%	21.80 ± 0.21	1.17 ± 0.12	5.35
N sources (0.25%)	Peptone-yeast extract (1:1)	22.00 ± 0.10	1.35 ± 0.24	6.13

Abbreviations: ND, not determined, to select the optimal temperature, the OD was measured.

**Table 4:** Effect of increasing substrate concentrations on the PHB bio-based polymer final yield.

Substrate Concentration g/L	DCW g/L	Bio-Based Polymer g/L	Bio-Based Polymer %
0.5	21.31 ± 0.23	0.92 ± 0.09	4.32
1	22.00 ± 0.10	1.35 ± 0.24	6.13
2	22.89 ± 0.15	1.38 ± 0.31	6.02
3	23.77 ± 0.24	1.54 ± 0.19	6.45
4	24.62 ± 0.11	1.22 ± 0.26	4.96
5	24.89 ± 0.17	1.04 ± 0.30	4.18

## Data Availability

The data presented in this study are available on request from the corresponding author.

## Supplementary Materials

The following supporting information can be downloaded at: https://www.mdpi.com/article/10.3390/polym15030512/s1. Figure S1: Alignment of 1b1 16S fragment (query) against Sphingomonas dokdonensis (sbjct), the best hit of BlastN output. Vertical lines indicate matching bases.
