# Supplementary material for: A Novel Sphingomonas sp. Isolated from Argan Soil for the Polyhydroxybutyrate Production from Argan Seeds Waste
Source: Polymers (Basel). 2023 Jan 18;15(3):512. doi: 10.3390/polym15030512 (PMC9921021; doi:10.3390/polym15030512)
Supplement: Supplementary file 1 [file polymers-15-00512-s001.zip › polymers-2125926-supplementary.pdf]

Supplementary Materials

A Novel *Sphingomonas* sp. Isolated from Argan Soil for the Polyhydroxybutyrate Production from Argan Seeds Waste

Amina Aragosa [1,2], Benedetta Saccomanno [1], Valeria Specchia [1] and Mariaenrica Frigione [3,*]

[1] Department of Biological and Environmental Sciences and Technologies, University of Salento, 73100 Lecce, Italy
[2] School of Science and Engineering, Al Akhawayn University, Ifrane 53000, Morocco
[3] Department of Innovation Engineering, University of Salento, 73100 Lecce, Italy
* Correspondence: mariaenrica.frigione@unisalento.it; Tel.: +39-0832-297215

Score	Expect	Identities	Gaps	Strand
1118 bits(605)	0.0	623/632(99%)	0/632(0%)	Plus/Plus

```
Query  1    ATGACTGCTAATACCGGATGATGACCTTAAGGCCAAAGATTTATCGCCCGAGGATGAGCC  60
            ||||||||||||||||||||||||| ||||| ||||||||||||||||||||||||||||
Sbjct  100  ATGACTGCTAATACCGGATGATGACGTTAAGTCCAAAGATTTATCGCCCGAGGATGAGCC  159

Query  61   CGCGTAAGATTAGCTAGTTGGTGTGGTAAGAGCGCACCAAGGCGACGATGCTTAGCTGGT  120
            |||||| |||||||||||||||||||||||||||||||||||||||||| ||||||||||
Sbjct  160  CGCGTAGGATTAGCTAGTTGGTGTGGTAAGAGCGCACCAAGGCGACGATCCTTAGCTGGT  219

Query  121  CTGAGAGGATGATCAGCCCCACTGGGACTGAGACACGGCCCAAACTCCTACGGGAGGCAG  180
            |||||||||||||||||| ||||||||||||||||||||||| |||||||||||||||||
Sbjct  220  CTGAGAGGATGATCAGCCACACTGGGACTGAGACACGGCCCAGACTCCTACGGGAGGCAG  279

Query  181  CAGTGGGGAATATTGGACAATGGGCGAAAGCCTGATCCAGCAATGCCGCGTGAGTGATGA  240
            ||||||||||||||||||||||||||||||||||||||||||||||||||||||||||||
Sbjct  280  CAGTGGGGAATATTGGACAATGGGCGAAAGCCTGATCCAGCAATGCCGCGTGAGTGATGA  339

Query  241  AGGCCTTAAGGTTGTAAAGCTCTTTTACCCGGGATGATAATGACAGTACCGGGAGAATAA  300
            |||||||| |||||||||||||||||||||||||||||||||||||||||||||||||||
Sbjct  340  AGGCCTTAGGGTTGTAAAGCTCTTTTACCCGGGATGATAATGACAGTACCGGGAGAATAA  399

Query  301  GCTCCGGCTAACTCCGTGCCAGCAGCCGGGGTAATACGGAGGGAGCTAGCGTTGTTCGGA  360
            |||||||||||||||||||||||||||| |||||||||||||||||||||||||||||||
Sbjct  400  GCTCCGGCTAACTCCGTGCCAGCAGCCGCGGTAATACGGAGGGAGCTAGCGTTGTTCGGA  459

Query  361  ATTACTGGGCGTAAAGCGCACGTAGGCGGCTTTGTAAGTTAGAGGTGAAAGCCTGGAGCT  420
            ||||||||||||||||||||||||||||||||||||||||||||||||||||||||||||
Sbjct  460  ATTACTGGGCGTAAAGCGCACGTAGGCGGCTTTGTAAGTTAGAGGTGAAAGCCTGGAGCT  519

Query  421  CAACTCCAGAATTGCCTTTAAGACTGCATCGCTCGAATCCAGGAGAGGTGAGTGGAATTC  480
            ||||||||||||||||||||||||||||||||||||||||||||||||||||||||||||
Sbjct  520  CAACTCCAGAATTGCCTTTAAGACTGCATCGCTCGAATCCAGGAGAGGTGAGTGGAATTC  579

Query  481  CGAGTGTAGAGGTGAAATTCGCAGATATTCGGAAGAACACCAGTGGCGAAGGCGGCTCAC  540
            ||||||||||||||||||||| ||||||||||||||||||||||||||||||||||||||
Sbjct  580  CGAGTGTAGAGGTGAAATTCGTAGATATTCGGAAGAACACCAGTGGCGAAGGCGGCTCAC  639

Query  541  TGGACTGGTATTGACGCTGAGGTGCGAAAGCGTGGGGAGCAAACAGGATTAGATACCCTG  600
            ||||||||||||||||||||||||||||||||||||||||||||||||||||||||||||
Sbjct  640  TGGACTGGTATTGACGCTGAGGTGCGAAAGCGTGGGGAGCAAACAGGATTAGATACCCTG  699

Query  601  GTAGTCCACGCCGTAAACGATGATAACTAGCT  632
            ||||||||||||||||||||||||||||||||
Sbjct  700  GTAGTCCACGCCGTAAACGATGATAACTAGCT  731
```

Figure S1. Alignment of 1b1 16S fragment (query) against Sphingomonas dokdonensis (sbjct), the best hit of BlastN output. Vertical lines indicate matching bases.
